# Permissiveness of firearm laws, pro-gun culture, and suicides by firearm in the U.S., 2000–2016

**DOI:** 10.1016/j.puhip.2021.100218

**Published:** 2021-11-15

**Authors:** Abhery Das, Parvati Singh, Tim Bruckner

**Affiliations:** a Program in Public Health, University of California, Irvine, USA; b Division of Epidemiology, College of Public Health, The Ohio State University, USA; c Center for Population, Inequality, and Policy, University of California, Irvine, USA

**Keywords:** Suicide, Firearm policy, Suicide prevention, Gun culture, (NAPHSIS), National Association for Public Health Statistics and Information Systems, (STROBE), Strengthening Reporting of Observational Studies in Epidemiology, (NRA), National Rifle Association, (ERPOs), Extreme Risk Protection Orders

## Abstract

**Objectives:**

Stricter firearm policies correlate with lower suicides by firearm in the US. However, much work examines policies in isolation and does not investigate firearm policies as they relate to US pro-gun culture. We examine the relation between permissiveness of state firearm laws, gun culture, and suicides by firearm.

**Study design:**

Panel longitudinal study.

**Methods:**

The count of suicides by firearm for 50 US states from 2000 to 2016 served as the outcome. Permissiveness of multiple state firearm laws, based on ratings from the Traveler's Guide to the Firearm Laws of the Fifty States, served as the exposure. These ratings, measured at the state-year, capture not only the overall policy environment but also the extent to which the state exhibits a pro-gun culture. We applied a fixed effects negative binomial count model, which controls for the population-at-risk, to examine suicides overall and by race/ethnicity and gender.

**Results:**

A 10-unit increase in permissiveness of state firearm laws corresponds with 2% greater suicides by firearm overall (Incidence rate ratio [IRR] = 1.02; 95% CI: 1.01–1.03) and among non-Hispanic white males ([IRR] = 1.02, 95% CI: 1.01–1.02).

**Conclusions:**

Findings, if replicated, indicate that states enacting more restrictive firearm policies, and lessening a pro-gun culture, may lead to reductions in suicide by firearm.

## Highlights

• Firearms persist as the most common method of suicide in the US.
• Firearm policies and the broader pro-gun culture may correspond with suicides.
• Permissiveness of state firearm laws precede increases in suicides by firearm.
• A restrictive suite of firearm policies may reduce suicides by firearm.

## 1 Introduction

The suicide rate in the U.S. increased by more than 30% from 1999 to 2017. In 2018, 48,344 Americans died by suicide [1]. As the tenth leading cause of death, suicide remains most prevalent among Non-Hispanic whites [2]. Men face a greater risk of suicide death than do women, accounting for 69% of all suicides in 2017 [3]. Men choose more lethal methods of suicide, such as firearms, relative to women [4]. However, firearms persist as the most common method of suicide in the U.S. across both genders [4].

For many, impulsivity plays a role in suicide completion. An estimated 24% of people take less than 5 min between making the decision and attempting suicide. An estimated 70% take under an hour [5,6]. Access to firearms, especially during periods of crisis, increases risk of suicide death [7]. International and national organizations assert that policies and interventions restricting access to guns serves as a tool for suicide prevention [8,9]. This view coheres with the notion that at least a subset of persons intending to commit suicide by firearm, but who have limited access to one, do not commit suicide by other means.

More than 10 case-control studies find that those dying by suicide had a higher likelihood of having a firearm in their household [5]. In addition to individual-level studies, ecological studies find that states with higher gun ownership correspond with higher rates of suicide by firearm [10,11]. State-level restrictions in firearm policy may also affect the rate of suicide by firearm. U.S. federal policy, under the Brady Handgun Violence Prevention Act, requires background checks for firearm purchases with licensed firearm dealers. However, almost all other firearm policies vary by state [12]. Working within the federal framework, states retain the power to regulate possession, transfer, and use of firearms [12].

Several longitudinal studies report that states with restrictive firearm policy also exhibit fewer suicides by firearm [7,[13], [14], [15], [16], [17], [18]]. The laws examined include permit-to-purchase handguns, youth focused firearm laws, safer storage, and handgun waiting periods [[13], [14], [15]]. This work generally supports that state policies may serve as an important tool for preventing suicides by firearm.

The literature, while important, remains limited in three significant ways. First, states enact a multitude of laws and regulations that affect gun restrictions. Some examples include permit and license restrictions, background checks, gun seizures among persons deemed a danger to self or others, open/concealed carry at various locations, ammunition possession, semi-automatic/high-capacity magazines/machine gun laws, safer storage, and ownership declaration to law enforcement. Examination of each policy in isolation ignores the ecological context in which many policies create a broader policy landscape regarding firearm restrictions. Second, previous work has not investigated suicides by firearm in conjunction with firearm policies and a broader pro-gun culture.

Scholars have defined gun culture as encompassing how individuals and institutions consciously and unconsciously interact with firearms, through beliefs, thoughts, behavior, social and legal norms, as well as the social structures they project onto them [19]. Coined in 1970, the term gun culture describes Americans' unique belief in that the people's right to bear arms remains the greatest protection of their individual rights and a safeguard of democracy [20]. Policies, including stand-your-ground laws and assault weapon bans, have historically influenced pro-gun culture within a state [19]. Evaluating the permissiveness of firearm policies, as they relate to pro-gun culture, may allow for a more comprehensive understanding of the ease of firearm access, availability, and use within a state and its relation to suicides by firearm.

Policies, including stand-your-ground laws and assault weapon bans, have historically influenced pro-gun culture within a state [19]. Evaluating the restrictiveness of firearm policies, as they relate to pro-gun culture, may allow for a more comprehensive understanding of the ease of firearm access, availability, and use within a state and its relation to suicides by firearm. Firearm permissiveness, therefore, lies at the intersection of firearm policy and pro-gun culture.

Third, much work does not examine potential racial/ethnic and gender differences in response to policies on firearm permissiveness and suicides by firearm. In 2017, 49% of non-Hispanic white households owned a firearm, more than any other race/ethnicity in the US [21]. Non-Hispanic whites, along with American Indian/Alaska Natives, also had the highest incidence of suicides [2]. Additionally, non-Hispanic white males accounted for 69.7% of suicide deaths in 2017 [3]. Race/ethnic groups differ in their access to behavioral health treatment, levels of income inequality, as well as other societal factors [22,23]. Investigating the association between permissive firearm policies and suicides by firearm by race/ethnicity and gender may allow for effectively directing suicide prevention efforts within states.

We address these limitations and test the hypothesis that state-level increases in firearm permissiveness correspond with an increase in suicides by firearm. We also investigate this potential relation by race/ethnicity and gender. We examine 319,919 suicides by firearm in 50 states from 2000 to 2016, a period coinciding with the increase in suicide rates in the US.

## 2 Methods

### 2.1 Study population

We used de-identified, publicly available data for suicides by firearm from the National Vital Statistics System Underlying Cause of Death Files for 2000–2016 [24]. We retrieved these data from The National Association for Public Health Statistics and Information Systems (NAPHSIS), a federal non-profit, that makes files publicly available through an application process [24]. NAPHSIS represents state vital records and public health statistics offices in the U.S. with 250 public health professionals from each of the US states [25]. We followed the Strengthening Reporting of Observational Studies in Epidemiology (STROBE) reporting guidelines for this study [26]. The University of California, Irvine, institutional review board deemed this study exempt owing to the use of publicly available, deidentified data.

### 2.2 Outcome variable

We retrieved suicide data from the 2000–2016 National Vital Statistics System Underlying Cause of Death Files [25]. We, consistent with the literature, identified suicide by firearm deaths using ICD-10 codes X72- X74 for “Intentional self-harm (suicide) by discharge of firearms” [24]. We extracted the number of suicides by firearm from all U.S. states from 2000 to 2016. We examined a 17-year period coinciding with the rapid rise of suicide rates across the country [27].

### 2.3 Exposure variable

We obtained ratings for U.S. firearm laws, by state, from the 2000–2016 editions of the Traveler's Guide to the Firearm Laws of the Fifty States [28]. Reeping et al. previously utilized this measure to evaluate state firearm laws and mass shootings [29]. The Guide provides annual ratings of the permissiveness and restrictiveness of U.S. firearm laws in each state for gun owners traveling across state lines [28]. The Guide outlines firearm regulations and provides a composite score between 0 (completely restrictive) to 100 (completely permissive) for each of the 50 states [28]. The composite score comprises more than 13 factors related to regulations including permit and license restrictions, open/concealed carry at various locations (i.e., National Parks, restaurants, schools, hotels), stand-your-ground laws, ammunition possession, semi-automatic/high capacity magazines/machine gun laws, declaring ownership to law enforcement, and variation of firearm laws across the state [28].

The Guide summarizes state firearm policies in order for gun owners to avoid the legal consequences associated with having firearms. Endorsed by the National Rifle Association (NRA), it provides a pro-gun and anti-control measure for firearm policies, as opposed to more traditional sources such as Giffords Law Center [30]. At the intersection of firearm policy and pro-gun culture, the Guide incorporates policies such as stand-your-ground and machine gun laws which have previously been associated with measures of pro-gun culture [19].

### 2.4 Control variables

Given that broader structural factors remain strong predictors of mental health disorders, suicidality, and violent behavior, we included as control variables the Gini index (a measure of income inequality) and percent of the population below the federal poverty line [[31], [32], [33], [34]]. We also included percent of the population with a high school diploma/GED to capture socioeconomic predictors of suicidality [33,35]. Next, we used as a control variable state-level per capita mental health expenditure [[36], [37], [38]]. Lastly, we obtained population estimates for each state by race/ethnicity and gender from the U.S. Census Bureau Population Estimates for 2000 to 2016 [50].

### 2.5 Statistical analysis

Our final analytical sample comprised 319,919 suicides by firearm, among all race/ethnicities and genders, from 50 states in the US from 2000 to 2016. For each state, we linked firearm law ratings to race and gender-specific suicide by firearm counts and other state-level covariates. This process yielded a sample of 8500 “state-years” (i.e., 8500 = 50 states * 17 years * 2 genders * 5 races).

State permissiveness of firearm law ratings serve as the exposure variable, while suicides by firearm counts served as the outcome variable. Because our outcome variable is a count of suicide by firearm deaths, we employed a negative binomial regression approach using as the offset (i.e., at-risk denominator) the state's annual population estimates by race/ethnicity and gender. This specification accounts for population changes over time by race/ethnicity and gender, while also examining suicides by firearm as proportional to population size. The negative binomial specification has widespread use in literature concerning suicides [39,40]. This approach also provided a better model fit than a Poisson regression given overdispersion of count of suicides by firearm.

Suicides by firearm may vary substantially across states and years for reasons unrelated to permissiveness of state firearm laws. Omitted state-level variables that remain stable over time may bias effect estimates if they correlate with permissiveness of state firearm laws or suicides by firearm. To control for this potential bias, we included state fixed effects. This approach permits estimation of within-state change in the outcome as a function of change in the exposure variable. We also specified year fixed effects to control for year-specific factors (e.g., the 2008 economic recession) that may correspond with an increase or decrease in suicides. The state and year fixed effects model allows examination of the year-to-year changes in suicides by firearm in a state as a function of year-to-year changes in permissiveness of state firearm laws.

In addition, we controlled for state-level attributes that may correlate with permissiveness of state firearm laws or suicides by firearm. These variables included the Gini index, percent of the population below the federal poverty line, percent of the population graduated from high school, and per capita mental health expenditure. Given the strong patterning of suicide mortality by race and gender in the US, we also performed stratified analyses to examine whether permissiveness of state firearm laws corresponded with an increase in suicides by firearm by race/ethnicity and gender [1]. As a sensitivity analysis, we incorporated additional state-level covariates that may also influence suicides by firearm: the unemployment rate and population without health insurance [[41], [42], [43], [44]]. We performed all analyses using Stata SE version 16.0. We interpreted estimates with a 2-sided p-value < 0.05 as statistically detectable.

## 3 Results

**Fig. 1:**
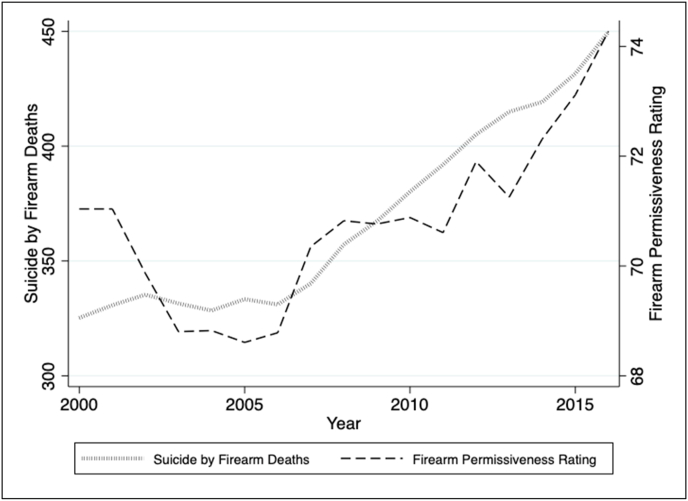
Average suicide by firearm deaths and firearm permissiveness ratings in the 50 US states, 2000–2016.

Over the test period, suicides by firearm deaths average 376.20 per state-year from 2000 to 2016 (Table 1). Firearm permissiveness averages 72.08 per state-year on a scale of 0 (completely restrictive laws) to 100 (completely permissive laws). Suicides by firearm increase steadily from 2000 to 2016 (Fig. 1), which coheres with the concurrent rise in overall suicide rates during that time period [2]. Firearm permissiveness also increases over the study period with a slight decrease before 2005. Firearm permissiveness ratings show variation across the study period with greater variation in the most restrictive firearm policy states (Supplement Figure 1). State-year suicide death counts (after stratifying by five race/ethnicities and two genders) showed a highly skewed distribution (Supplement Figure 2), which coheres with literature on the rarity and episodic nature of suicide. For this reason, we used negative binomial regression analysis.

**Table 1:** Summary statistics of suicides by firearm, state firearm laws, and structural and socio-economic covariates in 50 US states, 2000–2016.

Variable	Mean	Standard Deviation
Suicide by firearm deaths	376.20	349.02
Permissiveness of state firearm laws	72.08	23.48
Population at <200% of the federal poverty line (%)	14.44	3.98
Population graduated from high school (%)	64.19	3.97
Per capita mental health expenditure ($)	677.97	1012.85
Gini index	0.60	0.04

Table 2 shows fixed effects negative binomial regression Results in which a 10-unit increase in permissiveness of state firearm laws varies with a 2% increase in suicides by firearm (Incidence rate ratio [IRR] = 1.02; 95% CI, 1.01–1.03). Results stratified by gender and race/ethnicity indicate that a 10-unit increase in permissiveness of state firearm laws corresponds with a 2% increase in suicides by firearm among non-Hispanic white males (IRR = 1.02; 95% CI, 1.01–1.02) (Table 3, Model A). Suicides by firearm vary positively with permissiveness of state firearm laws for non-Hispanic white females, although not reaching conventional levels of statistical detection (IRR = 1.02; 95% CI, 0.99–1.04) (Table 3, Model D). We do not reject the null for any other group.

**Table 2:** Fixed effects negative binomial regression results predicting Incidence Rate Ratio (IRR) of suicides by firearm as a function of 10-unit increments in permissiveness of state firearm laws across the 50 US states, 2000–2016.

Covariates	IRR	95% CI
Permissiveness of state firearm laws	1.02 ****	1.01–1.03
Population at <200% of the federal poverty line (%)	0.99	0.99–1.01
Population graduated from high school (%)	0.99	0.99–1.00
Per capita mental health expenditure ($)	0.99 ****	0.99–0.99
Gini index	0.93	0.66–1.29
Race/Ethnicity (reference: other race/ethnicities^a^)		
White^a^	3.43 ****	3.31–3.56
Black American^a^	1.23 ****	1.18–1.28
Hispanic	0.82 ****	0.78–0.85
Gender (reference: Female)		
Male	7.06 ****	6.95–7.17
N	8500	

*p < 0.1, **p < 0.05, ***p < 0.01, ****p < 0.001.

Year indicator variables included, but not shown.

a Non-Hispanic

**Table 3:** Fixed effects negative binomial regression results predicting Incidence Rate Ratio (IRR) of suicides by firearm as a as a function of 10-unit increments in permissiveness of state firearm laws across the 50 US states, 2000–2016, for males and females by race/ethnicity.

Covariates	Model (a) Male Whites^a^		Model (b) Male Black Americans^a^		Model (c) Male Hispanic		Model (d) Female Whites^a^		Model (e) Female Black Americans^a^		Model (f) Female Hispanic	
	IRR	95% CI	IRR	95% CI	IRR	95% CI	IRR	95% CI	IRR	95% CI	IRR	95% CI
Permissiveness of state firearm laws	1.02 ****	1.01–1.02	1.01	0.99–1.04	1.01	0.99–1.04	1.02 *	0.99–1.04	0.99	0.98–1.04	1.03	0.99–1.15
Population at <200% of the federal poverty line (%)	0.99	0.99–1.00	1.00	0.98–1.03	1.02	0.99–1.04	0.99	0.98–1.01	1.05	0.98–1.12	1.02	0.94–1.09
Population graduated from high school (%)	0.99	0.99–1.00	0.99	0.98–1.01	1.00	0.99–1.19	0.99 *	0.98–1.00	0.96 *	0.92–1.00	0.99	0.94–1.04
Per capita mental health expenditure ($)	1.00	0.99–1.00	1.00	0.99–1.00	1.00	0.99–1.00	1.00	0.99–1.00	1.00	0.99–1.00	1.00**	0.99–0.99
Gini index	1.04	0.77–1.40	1.60	0.61–4.19	0.97	0.28–3.38	0.77	0.39–1.54	3.85	.21–70.32	15.35	.32–736.87
N	850		850		850		850		765		816	

p < 0.1, **p < 0.05, ***p < 0.01, ****p < 0.001.

American Indian/Alaska Native and Asian/Pacific Islander Results available upon request

Year indicator variables included, but not shown.

a Non-Hispanic

Results from our sensitivity analysis, incorporating additional state-level covariates, remain similar to our original test (Supplemental Table 1). We also used the *margins* command in Stata to estimate the total number of suicides statistically attributable to less restrictive firearm policies (Supplemental Fig. 3). Applying this prediction to the total number of state-year groups analyzed, we find approximately 168 additional suicides by firearm statistically attributable to every ten-unit increase in permissiveness of state firearm laws.

## 4 Discussion

Firearms account for more than half of all suicide deaths [1]. Impulsivity plays a role in many suicide attempts. Greater firearm use and access, by way of permissive firearm laws, may influence the decision to attempt suicide [5,6]. In this study, we examine the relation between permissiveness of state firearm laws and suicides by firearm across 50 US states from 2000 to 2016. We find that an increase in the permissiveness of state firearm laws corresponds with an increase in suicides by firearm. This relation concentrates among white men, with a modest increase for white women. Permissiveness of state firearm laws may increase suicides by firearm among whites but not in other racial/ethnic groups.

In the US, whites have the highest rates of both suicide and firearm ownership [1,21]. Among Americans owning a firearm, a majority (67%) own them for protection [21]. Although we do not know whether Americans owning firearms for protection later die by firearm suicide, literature finds that increased firearm ownership corresponds with increased suicides by firearm [10]. This result warrants further investigation as to whether the intent of firearm ownership later predicts suicide by firearm.

Previous literature also reports that individual policies for permit to purchase, safer storage, background checks, and extreme risk protection orders (ERPOs) correspond with decreased suicides by firearm [13,14,45]. However, individual policies do not capture the overall policy landscape within a state. As our findings indicate, a suite of policies attributed to pro-gun culture within a state, such as stand-your-ground and machine gun laws, also correspond with suicides by firearm. The NRA's pro-gun and anti-control advocacy efforts have made a substantial influence on American firearm culture with over 5 million members across the country [19]. Firearms have become a matter of liberty and personal responsibility with 75% of Americans believing the 2^nd^ amendment (“The right to keep and bear arms”) remains essential to their own sense of freedom [46]. Endorsed by the NRA, the Traveler's Guide to Firearm Laws in the Fifty States provides a pro-gun perspective on firearm policy, as opposed to more traditional sources.

Our results find that permissiveness of firearm laws does not correspond with suicides by firearm in other race/ethnicity groups. Given the recent rise in Black American youth suicide, further research needs to examine whether and to what extent firearm laws correspond with suicides in this age group [47]. Firearms account for more than 52% of male Black American youth suicides and 21% of female Black American youth suicides [47]. However, given that youth cannot buy firearms legally and that five out of six firearms recovered by law enforcement classify as illegal, an exploration of the illegal firearm market may provide more insight into youth suicide by firearm rates [48].

## 5 Strengths and limitations

Strengths of our analyses include the use of a comprehensive national database of all suicide by firearm deaths. Additionally, we utilized permissiveness of state firearm law ratings from the Traveler's Guide to the Firearm Laws of the Fifty States, a source endorsed by the National Rifle Association. Written for gun owners, the Guide captures the ease of using and accessing firearms by comprising a multitude of state firearm laws, as they relate to pro-gun culture. Previous literature has also used this variable and documented a positive relation between firearm permissiveness and mass shootings in the US [29]. Such work emphasizes the role of firearm permissiveness, at the intersection of firearm policy and pro-gun culture, and the legal landscape's influence on various forms of firearm violence.

Our study period (2000–2016) coincides with the rise in suicide rates in the US. Longitudinal analyses spanning 17 years allowed us to incorporate state and year fixed effects that control for time-invariant unobserved state attributes that may correspond with suicides by firearm. We also controlled for larger societal and structural factors, used in previous suicide and firearm policy literature, that may affect suicides by firearm, such as income inequality, percent of the population below the federal poverty line, percent of the population that graduated from high school, and state expenditures for mental health [23,36,37].

Limitations include that we do not know how individuals dying from firearm suicide obtained or accessed firearms. State-level analysis does not allow us to draw conclusions about individuals in those states. These individuals may not own firearms even if they live in states with permissive firearm laws. However, we do know that individuals who die by firearm suicide had access to a firearm. In addition, we do not examine age-specific responses in suicides by firearm with respect to permissiveness of state firearm laws. Prior research provides some evidence of greater suicide by firearm mortality with increases in permissive firearm policies among males aged 55 years or greater [45]. Whereas we did not, *a priori*, hypothesize age-specific differences, future research may examine these relations in greater detail.

Our exposure also does not provide ratings for individual firearm policies within states. Although a cumulative rating may mask the influence of a particular policy, the score provides an understanding of the overall policy landscape, as it relates to pro-gun culture, within a state. Previous research has examined whether particular policies for safe storage or ERPOs correspond with suicides by firearm. However, further research should investigate how other aspects of pro-gun culture, such as NRA membership, may correspond with suicides by firearm.

## 6 Conclusion

The suite of firearm laws ranges widely—from concealed carry policies to regulations for automatic machine guns and carrying guns in schools. Taken together, the large set of policies creates large variability across states and over time in firearm access, availability, and use. This policy landscape, in our view, provides a more comprehensive understanding of the influence of firearm laws—and associated pro-gun culture—than do single policies in isolation. Our findings indicate that more restrictive laws have the potential to reduce suicides by firearm. Extensions to our work should consider the collateral consequences of such potential policy changes, such as the increased use of the illegal gun market or suicides by other methods. Given its rising prevalence in the US and the substantial life-years lost due to suicide, these and other avenues of research on this topic appear warranted.

## Funding

National Institute of Mental Health (1R21MH110815-01A1).

## Ethics approval

The University of California, Irvine, institutional review board deemed this study exempt owing to the use of publicly available, deidentified data.

## Disclosures

All authors declare: no support from any organization for the submitted work; no financial relationships with any organizations that might have an interest in the submitted work in the previous 3 years; no other relationships or activities that could appear to have influenced the submitted work. The authors declare they have no conflict of interest.

## Data availability

The state-level datasets used for the analysis and the statistical code are available from the corresponding author. All authors, external and internal, had full access to all of the data (including statistical reports and tables) in the study and can take responsibility for the integrity of the data and the accuracy of the data analysis.

## Declaration of competing InterestCOI

The authors declare that they have no known competing financial interests or personal relationships that could have appeared to influence the work reported in this paper.
