# Supplementary material for: Permissiveness of firearm laws, pro-gun culture, and suicides by firearm in the U.S., 2000–2016
Source: Public Health Pract (Oxf). 2021 Nov 15;2:100218. doi: 10.1016/j.puhip.2021.100218 (PMC9461642; doi:10.1016/j.puhip.2021.100218)
Supplement: Multimedia component 1 [file mmc1.docx]

**Supplemental Material**

**Supplement Figure 1.** Firearm permissiveness ratings^a^ by average rating quartile for each of the 50 US states, 2000-2016.

Panel 1

Panel 2

Panel 3

Panel 4

*^a^Firearm permissiveness rating ranges from 0 (completely restrictive) to 100 (completely permissive).*

**Supplemental Figure 2.** Frequency distribution of suicide by firearm deaths in 8,500 state-years after stratification by two genders and five race/ethnicities^a^ in the 50 US states, 2000-2016.

**
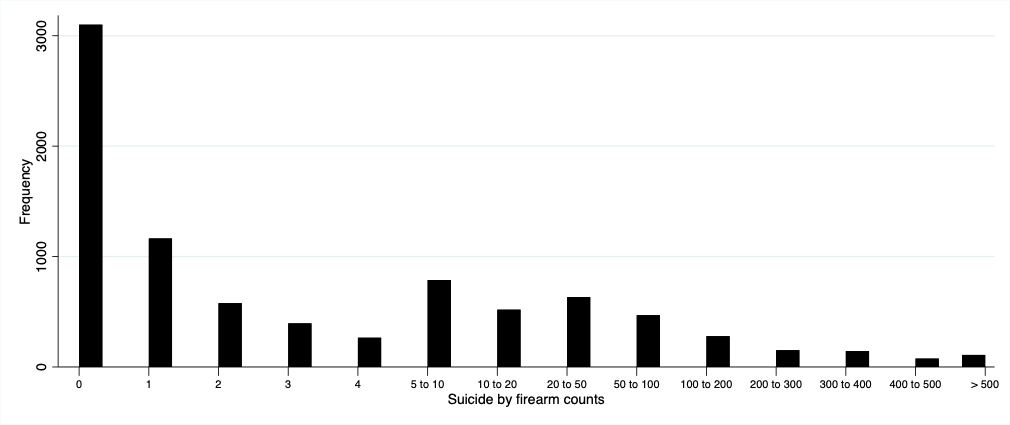
**

*^a^ 8,500 state year groups = 50 states * 17 years * 2 genders * 5 race/ethnicities*

**Supplemental Figure 3:** Average marginal increase (with 95% confidence intervals) in suicides by firearm, per 10-unit increase in permissiveness of state firearm laws.


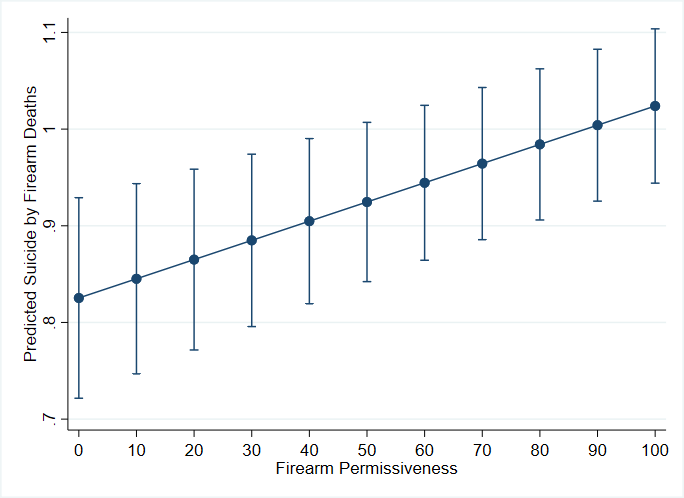


**Supplemental Table 1.** Fixed effects negative binomial regression results predicting Incidence Rate Ratio (IRR) of suicides by firearm as a function of 10-unit increments in permissiveness of state firearm laws, **including additional state-level covariates**, across the 50 US states, 2000-2016.

| **Covariates** | **IRR** | **95% CI** |
| --- | --- | --- |
| Permissiveness of state firearm laws | 1.02 ^****^ | 1.01-1.03 |
| Population at <200% of the federal poverty line (%) | 0.99 | 0.99-1.01 |
| Population graduated from high school (%) | 1.01 | 0.99-1.02 |
| Per capita mental health expenditure ($) | 0.99 ^****^ | 0.99-0.99 |
| Gini index | 0.73 | 0.99-0.99 |
| **Unemployment rate** | **1.01** | **0.99-1.01** |
| **Population without health insurance (%)** | **0.99** | **0.99-1.01** |
| Race/Ethnicity (reference: other race/ethnicities ^a^) | | |
| White ^a^ | 4.58^****^ | 4.41-4.75 |
| African American ^a^ | 1.50^****^ | 1.43-1.57 |
| Hispanic | 1.29^****^ | 1.21-1.37 |
| Gender (reference: Female) | | |
| Male | 6.83^****^ | 6.68-6.98 |
| N | 4,000 | |

^*^ p<0.1, ^**^ p<0.05, ^***^p<0.01, ^****^p<0.001

^a^ non-Hispanic

*Year indicator variables included, but not shown*
